# Finding the Location of Axonal Activation by a Miniature Magnetic Coil

**DOI:** 10.3389/fncom.2022.932615

**Published:** 2022-06-29

**Authors:** Hui Ye

**Affiliations:** Department of Biology, Quinlan Life Sciences Education and Research Center, Loyola University Chicago, Chicago, IL, United States

**Keywords:** micromagnetic stimulation (μMS), unmyelinated axon, activation, activating function analysis, multi-compartment modeling, ion channels

## Abstract

Magnetic stimulation for neural activation is widely used in clinical and lab research. In comparison to electric stimulation using an implanted electrode, stimulation with a large magnetic coil is associated with poor spatial specificity and incapability to stimulate deep brain structures. Recent developments in micromagnetic stimulation (μMS) technology mitigates some of these shortcomings. The sub-millimeter coils can be covered with soft, biocompatible material, and chronically implanted. They can provide highly specific neural stimulation in the deep neural structure. Although the μMS technology is expected to provide a precise location of neural stimulation, the exact site of neural activation is difficult to determine. Furthermore, factors that could cause the shifting of the activation site during μMS have not been fully investigated. To estimate the location of axon activation in μMS, we first derived an analytical expression of the activating function, which predicts the location of membrane depolarization in an unmyelinated axon. Then, we developed a multi-compartment, Hodgkin-Huxley (H-H) type of NEURON model of an unmyelinated axon to test the impact of several important coil parameters on the location of axonal activation. The location of axonal activation was dependent on both the parameters of the stimulus and the biophysics properties of the targeted axon during μMS. The activating function analysis predicted that the location of membrane depolarization and activation could shift due to the reversal of the coil current and the change in the coil-axon distance. The NEURON modeling confirmed these predictions. Interestingly, the NEURON simulation further revealed that the intensity of stimulation played a significant role in the activation location. Moderate or strong coil currents activated the axon at different locations, mediated by two distinct ion channel mechanisms. This study reports several experimental factors that could cause a potential shift in the location of neural activation during μMS, which is essential for further development of this novel technology.

## Introduction

Magnetic stimulation for neural activation is widely used in clinical and lab research [reviewed in Ye and Kaszuba (2019)]. In comparison with electric stimulation with implanted electrodes, magnetic coils can provide transcranial stimulation by inducing electric current inside the brain (Walsh and Pascual-Leone, 2003; Ye and Steiger, 2015). This noninvasive method does not require the coil to be in direct contact with the target tissue (Maccabee et al., 1991, 1993; Ye et al., 2010, 2011; Ye and Steiger, 2015). This mitigates numerous problems that can arise at the brain-electrode interface, such as charge transfer, electrode surface modification, and corrosion (Polikov et al., 2005; Cogan, 2008; Koivuniemi et al., 2011). However, due to the large size of the coil and the fast decay of the induced electric field around it (Polk, 1990; Polk and Song, 1990), clinically employed coils cannot provide deep brain stimulation with high spatial resolution.

Recent developments in micromagnetic stimulation (μMS) technology significantly improved the specificity of coil stimulation (Bonmassar et al., 2012; Park et al., 2013). These miniature coils can be fabricated at the sub-millimeter scale. They are implantable under the cover of soft, biocompatible materials, which mitigates the cortical response to the implantation (Saxena et al., 2013; Canales et al., 2015), including inflammatory and immune responses caused by direct contact with the tissue (Kim et al., 2004; Lee et al., 2016; Liu et al., 2017). With such implantation, focal stimulation could be applied to the deep structure, and the stimulation intensity to the target tissue can be better controlled without causing significant side effects.

Neuromodulation effects of μMS have been reported in several recent studies. A commercially available miniature coil (inductor) has been used to activate individual retinal ganglion neurons with high amplitude pulses (Bonmassar et al., 2012). Application of this micromagnetic field to the dorsal cochlear nucleus activated the inferior colliculus neurons *in vivo* (Park et al., 2013). We reported that stimulation with the miniature coil using high-frequency pulses could block axonal conductance in unmyelinated axons (Skach et al., 2020) and in single-ganglion neurons (Ye and Barrett, 2021). Furthermore, the miniature coil can provide focal inhibition of epileptic form activity in the hippocampus of mice (Ye et al., 2020). The coil-induced electric fields can be specifically designed to activate some neuronal subpopulations, while simultaneously avoiding others (Lee et al., 2016; Golestanirad et al., 2018). Recent numerical studies demonstrated that the miniature coils were MRI compatible and produced minimal heating under MRI signals (Bonmassar and Serano, 2020).

Several studies investigated the axonal activation using μMS. Golestanirad et al. used a small inductor (coil) to activate axons in the dorsal cochlear nucleus (Golestanirad et al., 2018). Using a similar induction coil, Saha et al. activated the unmyelinated axons of CA3 neurons in the Schaffer collaterals to trigger synaptic transmission in the hippocampus (Saha et al., 2022). Lee et al. improved the shape design of the micro-coil to activate the apical dendrites of layer V pyramidal neurons (Lee et al., 2016; Lee and Fried, 2017). As a novel technology with significant clinical potential in the field of neuromodulation, the neural mechanism underlying μMS is largely unknown. Although the miniature coil is thought to provide excellent spatial specificity for neural activation, the exact location of neural activation and its dependency on the major coil parameters have not been fully investigated.

There are two approaches to estimate the location of neural activation by μMS: (1) activating function analysis and (2) numerical simulation using multi-compartment modeling.

The activating function analysis is based on the understanding that the gradients of the electric field along the neural tissue, or the *activating function* (Rattay, 1989), could be used to define the location and speed of membrane depolarization or hyperpolarization by extracellular stimulation (Rattay, 1986; Lee and Fried, 2017). As a quick and powerful engineering method, this analysis tool was frequently used to predict the location of the activation during neural stimulation. Lee et al. (2016) calculated the activating function in the three-dimensional space to estimate the location of cortical neuron activation and found that the gradient oriented normal to the cortical surface was the most important. Because the shape of the micro-coil directly affects the activating function, an activating function analysis was used to improve the design of the novel miniature coil with various shapes (Lee et al., 2019). Because the activating function analysis does not include membrane biophysics, such as the history of ion channel behavior, it assumes that the action potential will be initiated (i.e., axonal activation) at the location where the activating function is sufficiently large (Maccabee et al., 1993; Lee et al., 2019).

The multi-compartment modeling implements neurons that contain detailed ion channel properties and applies magnetic stimulations to the cell for activation. This allows the direct observation of neuron behavior under μMS. For example, a model of a myelinated axon was built to estimate the efficiency of the micro-coil-induced activation and its dependency on the coil orientation using the software package NEURON (Golestanirad et al., 2018). The authors demonstrated that the axon was the easiest to activate when the coil induces an electric field along the direction of the axon. To model neural activation under electric stimulation, researchers used a typical three-step simulation approach (Tai et al., 2005, 2009; Lu et al., 2008; Joucla et al., 2014). First, the electric current distribution generated by the coil was computed in three-dimensional (3D) space. Second, a multi-compartment model was constructed to represent the fine, geometric structure of the neuron, with channel mechanisms incorporated into each component. Finally, the electric field obtained from the first step was used to activate the membrane. This approach included detailed ion channel dynamics in the simulation, which was crucial to reveal the mechanisms underlying membrane activation. However, several assumptions were made to reduce the computation requirements. The extracellular electric field was usually computed without considering the existence of the tissue or its countereffect to the externally applied electric fields (Joucla et al., 2014; Ye and Steiger, 2015), which could introduce potential inaccuracies in the modeling (McIntyre et al., 2004; Lee and Grill, 2005).

To investigate the location of axonal activation and its dependency on the parameters that define the miniature coil stimuli, this study employed both the activating function analysis and the NEURON modeling approaches. We believe that the combined use of the two approaches, although each has its limitations, yields more reliable results than a singular method. The first part of the study derives an analytical expression of the activating function for a sub-millimeter circular miniature coil. Because the membrane depolarization does not necessarily guarantee excitation, the second part of the study simulates the location of axonal excitation using a multi-compartment NEURON model of an unmyelinated axon. The results reveal several key experimental factors that could cause the shifting of the activation site on the axon, therefore compromising the precision of neural stimulation by the miniature coil. Identification of these factors is essential for the further improvement of spatial resolution in neuromodulation with the novel μMS technology.

## Materials and Methods

### Calculation of Induced Electric Field Around a Circular Coil

**Figure 1:**
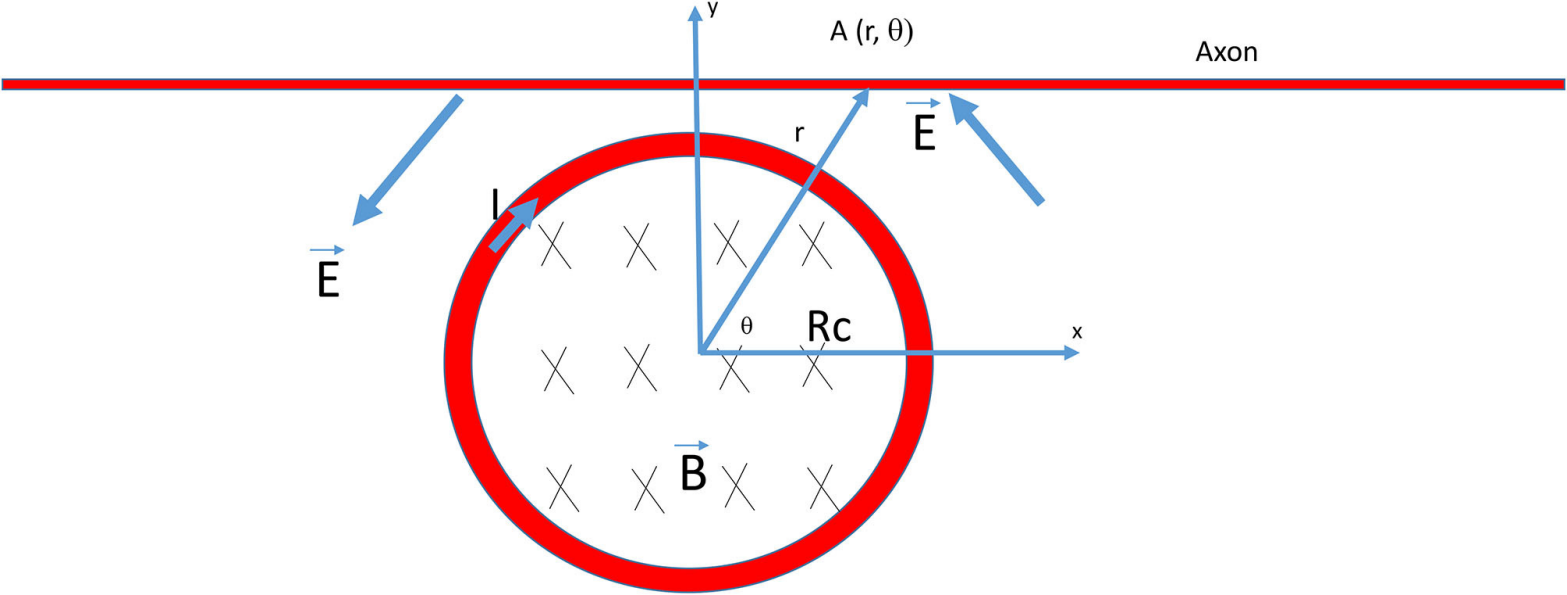
Electric field generated by a circular coil for the stimulation of an axon. The radius of the coil was R_c_. B was the magnetic flux generated by the current (*I*) inside the coil, which, in turn, induced an electric field (*E*) around the coil for axon stimulation. The rising phase of the coil current (*I*) generated a counterclockwise electric field (*E*) that traveled through the axon. The center of the coil was the origin of the polar system and A (r, θ) was a point on the axon.

The magnetic field generated by the miniature coil is given by the Faraday's law of induction,


(1) $$\begin{array}{l} \varepsilon =-\frac{d\emptyset_{B}}{dt} \end{array}$$

where ε is the electromotive force (EMF) and Φ_B_ is the magnetic flux. This can also be written in an integral form (Kelvin–Stokes theorem):


(2) $$\begin{array}{l} \oint \vec{E}\cdot d\vec{l}=-\iint \frac{\partial \vec{B}}{\partial t}\cdot d\vec{A} \end{array}$$

where $\vec{B}$ is the magnetic field inside the coil, $\vec{E}$ is the induced electric field, $\vec{dl}$ is an infinitesimal vector element or the path element, and $d\vec{A}$ is an infinitesimal vector element of area considered. Figure 1 illustrates the polar system whose center is overlapping with the center of the miniature coil. For a point A (r, θ) in this system, from Eq. (2), we obtain


(3) $$\begin{array}{l} E_{\theta }=-\frac{{R_{c}}^{2}}{2r}\frac{\partial B}{\partial t}\left(r>R_{c}\right) \end{array}$$

(4) $$\begin{array}{l} E_{r}=0\left(r>R_{c}\right) \end{array}$$

Here, *E*_θ_ is the $\vec{\theta }$ component of $\bar{E}$ and *E*_*r*_ is the $\vec{r}$ component. *R*_*c*_ is the radius of the coil and *r* is the distance between an arbitrary point and the center of the coil.

When a voltage pulse (V) is delivered to the miniature coil, it generates a magnetic field around it. The voltage across the coil is equal to the voltage drop due to the coil resistance and inductive impedance,


(5) $$\begin{array}{l} V=IR+L\frac{dI}{dt}, \end{array}$$

where *I* is the current in the coil, R is the coil resistance, and L is the inductance of the coil.

For the rising phase of the pulse, the solution of the coil current is


(6) $$\begin{array}{l} I=\frac{V}{R}\left(1-e^{-\frac{tR}{L}}\right) \end{array}$$

Therefore, *I* is zero at the beginning of the pulse and increases exponentially to a plateau value (V/R). For the falling phase of the pulse, the coil current is


(7) $$\begin{array}{l} I=\frac{V}{R}e^{-\frac{tR}{L}} \end{array}$$

Therefore, the coil current decays exponentially in the falling phase, from the maximum (V/R) to zero.

For a coil with a flowing current (*I*) inside, the magnetic field is calculated by


(8) $$\begin{array}{l} B=u_{0}\frac{NI}{l}=\frac{u_{0}NV}{Rl}\left(1-e^{-\frac{tR}{L}}\right) \end{array}$$

for the rising phase of the pulse, or


(9) $$\begin{array}{l} B=u_{0}\frac{NI}{l}=\frac{u_{0}NV}{Rl}e^{-\frac{tR}{L}} \end{array}$$

for the falling phase of the pulse, where N is the number of coil loops and *l* is the length of the coil.

Based on Lenz's law, the induced electric field is at its maximum at the onset of the square pulse, and then decays exponentially with time. Indeed, by combining Eqs. (3) and (8), the induced electric field outside the coil at the rising phase becomes


(10) $$\begin{array}{l} E_{\theta }=-\frac{{R_{c}\;}^{2}}{2r}\frac{Vu_{0}N}{Ll}e^{-\frac{tR}{L}} \end{array}$$

A similar analysis can be applied to the falling phase. By combining Eqs. (3) and (9),


(11) $$\begin{array}{l} E_{\theta }=\frac{{R_{c}\;}^{2}}{2r}\frac{Vu_{0}N}{Ll}e^{-\frac{tR}{L}} \end{array}$$

Therefore, the induced electric field is a biphasic signal. It is the largest at the onset and the offset (with opposite sign) of the stimulation pulse. It approaches zero along time following a relaxation course depending on constant parameters. In the above calculation, the model neglects the “secondary” magnetic field generated by the induced eddy current due to the relatively low electric conductivity of the tissue and the low frequency of the stimulus signal considered (Polk, 1990; Polk and Song, 1990).

### Measurement of Waveform of the Induced Electric Field

**Figure 2:**
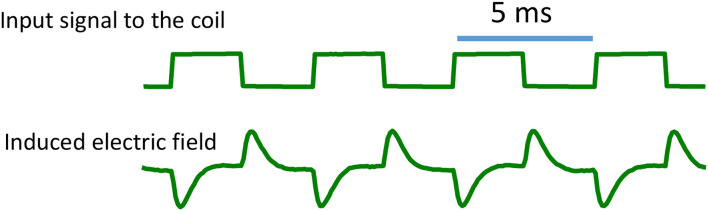
Induced electric field around the coil. When voltage pulses were delivered into the coil, they generated an electric field with a biphasic shape. The rising phase of the pulse generated a large electric field, followed by an exponential decay. The falling phase also generated a large electric field, but with reversed direction.

To validate the derivation of the biphasic shape of the magnetically induced electric field (Eqs. 10 and 11), we experimentally measured the induced electric field around the miniature coil using an electrophysiology setup. We filled a Petri dish with conductive saline and submerged the coil under the saline and delivered electric pulses to it using a signal generator and a power amplifier (Ye and Barrett, 2021). We positioned a glass electrode next to the coil to record the induced electric field. The recorded signal was then amplified by a model 1,700 differential AC amplifier (A-M Systems) and stored on a computer with the Spike 2 software (v. 7.2 Cambridge Electronic Design Limited). When a square pulse signal was delivered to the miniature coil, the shape of the induced electric field was biphasic (Figure 2). These measures were in agreement with that reported by Minusa et al. (2018), who found the similar biphasic shape generated by a stimulating pulse with 0.5-ms duration. In other studies, when square pulses were used for μMS, the electrophysiological electrode positioned next to the coil also picked up the biphasic signal during dorsal cochlear nucleus (Golestanirad et al., 2018) or cortical neuron (Lee and Fried, 2017) stimulations.

### Computation of Activating Function for the Miniature Coil

The gradients of the electric field along the axon define the location and speed of depolarization or hyperpolarization by the extracellular stimulation (Rattay, 1986; Lee and Fried, 2017). The component of the electric field gradient along the axon, or the activating function (Rattay, 1989), represents the driving force for activation of the axon. Therefore, it is possible to predict the location of neural stimulation with the activating function (Lee et al., 2016). Previously, we calculated the activating function for a monophasic signal (Skach et al., 2020). The following derivation considers the biphasic nature of the induced electric field, as both phases could play essential roles in axonal activation.

To derive the activating function for a miniature coil, the electric potential along the axon is first calculated (in the x direction, Figure 1).

Continued from Equation (11), the induced electric field is expressed on a Cartesian basis using the matrix transformation,


(12) $$\begin{array}{l} E\left(x,y\right)=\left[\begin{array}{cc} cos\theta & -sin\theta \\ sin\theta & cos\theta \end{array}\right]\left[\begin{array}{c} E_{r}\\ E_{\theta } \end{array}\;\right] \end{array}$$

where $\sin\theta =\frac{y}{r}$, $\cos\theta =\frac{x}{r}$, and *r* = (*x*^2^ + *y*^2^)^1/2^.

For the onset of the stimulation pulse, using Equations (10) and (12),


(13) $$\begin{array}{l} E_{x}=-\frac{Vu_{0}N{R_{c}\;}^{2}}{2Ll}\frac{y}{x^{2}+y^{2}}e^{-\frac{tR}{L}} \end{array}$$

(14) $$\begin{array}{l} E_{y}=\frac{Vu_{0}N{R_{c}\;}^{2}}{2Ll}\frac{x}{x^{2}+y^{2}}e^{-\frac{tR}{L}} \end{array}$$

For the offset of the pulse, using Equations (11) and (12),


(15) $$\begin{array}{l} E_{x}=\frac{Vu_{0}N{R_{c}\;}^{2}}{2Ll}\frac{y}{x^{2}+y^{2}}e^{-\frac{tR}{L}} \end{array}$$

(16) $$\begin{array}{l} E_{y}=-\frac{Vu_{0}N{R_{c}\;}^{2}}{2Ll}\frac{x}{x^{2}+y^{2}}e^{-\frac{tR}{L}} \end{array}$$

For the onset of the pulse, the activating function alone for the axon is


(17) $$\begin{array}{l} AF=\frac{\partial E_{x}}{\partial x}=-\frac{Vu_{0}N{R_{c}\;}^{2}}{Ll}x{y\left(x^{2}+y^{2}\right)}^{-2}e^{-\frac{tR}{L}} \end{array}$$

For the offset of the pulse, the activating function alone for the axon is


(18) $$\begin{array}{l} AF=\frac{\partial E_{x}}{\partial x}=\frac{Vu_{0}N{R_{c}\;}^{2}}{Ll}x{y\left(x^{2}+y^{2}\right)}^{-2}e^{-\frac{tR}{L}} \end{array}$$

The “neutral point” is a point on the axon where the activating function is zero. From Eqs. (17) and (18), this point is found to be located at x = 0. In Figure 1, if the coil current was in the counterclockwise direction and increasing over time (rising phase), the induced electric field had a clockwise direction. Points for peak depolarization and hyperpolarization are solved by $\frac{dAF}{dx}=0$. The point for peak depolarization is at


(19) $$\begin{array}{l} x=y/\sqrt{3} \end{array}$$

The location of peak hyperpolarization is at


(20) $$\begin{array}{l} x=-y/\sqrt{3}. \end{array}$$

Notably, these locations are defined by the coil's distance to the axon, suggesting that the location of polarization could be affected by the distance between the coil and the axon.

Finally, if the coil wind was perpendicular to the axon, and the axon was positioned in the z direction,


(21) $$\begin{array}{l} AF=\frac{\partial E}{\partial z}=0 \end{array}$$

Therefore, when the induced electric field is perpendicular to the axon, it is ineffective for axonal activation, as suggested by several simulation and experimental studies (Amassian et al., 1989; Basser and Roth, 2000; Golestanirad et al., 2018).

### Multi-Compartment NEURON Model of an Unmyelinated Axon Under Stimulation by a Miniature Coil

**Figure 3:**
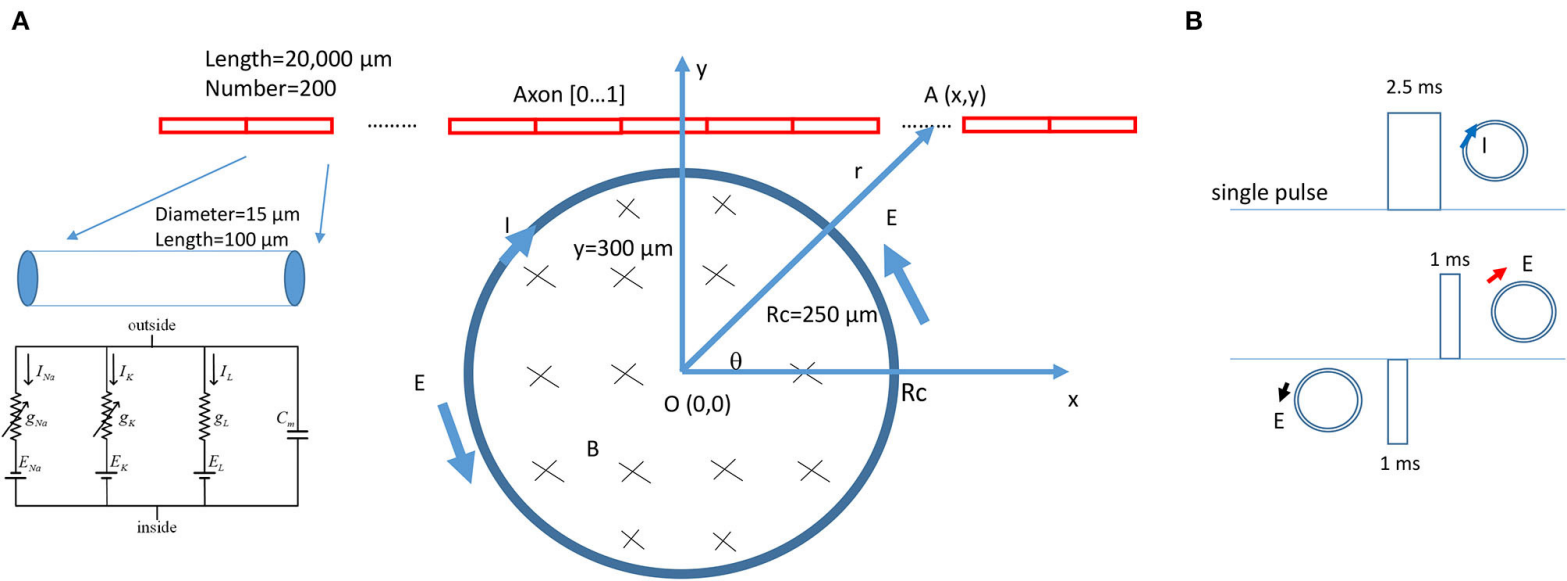
NEURON modeling of an unmyelinated axon under the stimulation of a circular miniature coil. **(A)** The multi-compartment model of the axon was 20,000 μm in length and was divided into 200 segments. Each segment was a cylinder of length 100 μm and diameter 15 μm. H-H type of ionic channels were inserted into each segment. The center of the coil was at point O (0,0). Distance between a point A (x,y) on the axon and O (0,0) was r. The coil radius was Rc = 250 μm. A single electric pulse was delivered into the coil for axon activation. **(B)** Modeling of the induced electric field as a biphasic signal. For computational simplicity, the induced electric fields generated by the rising phase and falling phase were modeled as short pulses. The coil current (clockwise, blue arrow) generated the induced electric field. The rising phase generated a counterclockwise electric field (black arrow). The falling phase generated a clockwise electric field (red arrow). The induced electric field contained a 1-ms pulse with a gap of 1.5 ms, followed by a 1-ms pulse with a reversed polarity.

A multi-compartment, unmyelinated axon model (Figure 3A) was implemented using the NEURON (v7.8) simulation environment package (Hines and Carnevale, 1997). The model simulated the axon as a cylinder 20,000 μm in length and 15 μm in diameter. The axon was divided evenly into 200 node segments (Table 1). The Hodgkin-Huxley (H-H)-type dynamics of the fast sodium, slow potassium, and leakage channels in the membrane were inserted into the nodes (Hodgkin and Huxley, 1952).

**Table 1:** Geometric parameters of the NEURON model for an unmyelinated axon.

**Geometric parameter**	**Value**
Axon diameter	15 μm
Number of axon segments	200
Length of axon segments	100 μm
Total axon length	200,00 μm
Location of coil center (x_coil_)	0 μm
Distance of coil center to axon (y_coil_)	300–800 μm

The ionic current (I) at the n-th segment of the neuron was described as


(22) $$\begin{array}{l} I_{n}=g_{Na}m^{3}h\left(V_{n}-V_{Na}\right)+g_{k}n^{4}\left(V_{n}-V_{k}\right)+g_{L}\left(V_{n}-V_{L}\right) \end{array}$$

where V_Na_, V_K_, and V_L_ were the equilibrium membrane potentials for sodium, potassium, and leakage channels, respectively. g_Na_, g_k_, and g_L_ were the maximal conductances of Na, K, and leakage channels, respectively. m and h represented the activation and inactivation of the sodium channels, respectively, whereas n represented the activation of potassium channels. The evolution equations for variables m, h, and n were


(23) $$\begin{array}{l} \frac{dm}{dt}=\alpha_{m}\left(1-m\right)-\beta_{m}m \end{array}$$

(24) $$\begin{array}{l} \frac{dh}{dt}=\alpha_{h}\left(1-h\right)-\beta_{h}h \end{array}$$

(25) $$\begin{array}{l} \frac{dn}{dt}=\alpha_{n}\left(1-n\right)-\beta_{n}n \end{array}$$

where α_*m*_, β_*m*_, α_*h*_, β_*h*_, α_*n*_, and β_*n*_ are rate constants (Table 2). Detailed electrical parameters (Table 2) of the modeled axon were adapted from a published model of the unmyelinated axon in *Aplysia californica* (Skach et al., 2020).

**Table 2:** Electric parameters of the NEURON model for an unmyelinated axon.

**Electrical parameter**	**Value**
Membrane capacitance (*C*_m_)	1 μF/cm^2^
*Fast Na+ channels*	
Max. sodium conductance (g_Na__) in the axon	0.12 S/cm^2^
Activation term (α_m_) of m gates	–(0.1ν + 4)(exp(−0.1ν – 4) – 1)^−1^
Inactivation term (β_m_) of m gates	4exp (–(ν+65)/18)
Time constant (t_m_) of m gates	3$\left(\alpha_{\text{m}}+\beta_{\text{m}}\right)*3{\left(t/10-2.0\right)}^{-1}$
Activation term (α_h_) of h gates	0.07exp(−0.05ν – 3.25)
Inactivation term (β_h_) of h gates	1/(exp(−0.1ν – 3.5) + 1)
Time constant (t_h_) of h gates	1.7 ${\left[\left(\alpha_{\text{h}}+\beta_{\text{h}}\right)*3\left(t/10-2.0\right)\right]}^{-1}$
Reversal potential (*E*_Na_)	50 mV
*Slow K+ channels*	
Max. conductance (g_K__) in the axon	0.036 S/cm^2^
Activation term (α_n_) of n gates	–(0.01ν + 0.55) (exp(−0.1ν – 5.5) – 1)^−1^
Inactivation term (β_n_) of n gates	0.125exp(–(ν +85)/80)
Time constant (t_n_) of n gates	5.6${\left[\left(\alpha_{\text{n}}+\beta_{\text{n}}\right)*3\left(t/10-2.0\right)\right]}^{-1}$
Reversal potential (*E*_K_)	−77 mV
*Leakage channels*	
Conductance (*g*_L_)	0.00028 S/cm^2^
Reversal potential (*E*_L_)	−65 mV

*t: environmental temperature in degrees Celsius*.

*v: membrane potential of a neural segment*.

### Incorporating the Coil With the Axon Model for Micromagnetic Stimulation

The potential differences between two points on the axon were calculated by integrating the scalar component of the electric field (Eqs. 15 and 16) along the path of the axon. For the onset of the pulse, the electric potential distribution along the axon was expressed as


(26) $$\begin{array}{l} V\left(x\right)=\int E_{x}\left(x\right)dx=-\frac{Vu_{0}N{R_{c}\;}^{2}}{2Ll}atan\left(\frac{x}{y}\right)e^{-\frac{tR}{L}} \end{array}$$

For the offset of the pulse, the electric potential distribution along the axon was expressed as


(27) $$\begin{array}{l} V\left(x\right)=\int E_{x}\left(x\right)dx=\frac{Vu_{0}N{R_{c}\;}^{2}}{2Ll}atan\left(\frac{x}{y}\right)e^{-\frac{tR}{L}} \end{array}$$

During NEURON simulation, the miniature coil was positioned at the middle point of the axon, where the center of the coil was 300–800 μm away from the axon (Figure 3A). The electric voltages induced by the miniature coil were calculated (Eqs. 26 and 27) and used to create the extracellular stimuli. A single pulse, 2.5 ms in duration, was programmed and applied to the coil for axon activation. Precise modeling of the exponential rise or decay of the induced electric voltage is computationally challenging. To simulate the waveform of the induced electric field in NEURON, we used biphasic short pulses with alternating direction to represent the induced electric field (Figure 3B). The duration of the pulse was 1 ms, as measured experimentally (Figure 2). During simulation, a vector was created to store the waveform of the stimulation for each time step. The current value of the extracellular potential was updated using “e_extracellular” at each compartment using the vector class' “play” method (Joucla et al., 2014). The model was ran at room temperature (20 °C) to simulate the environment temperature of the modeled unmyelinated axons [from marine mollusk *Aplysia californica* (Skach et al., 2020)]. The resting membrane potential was set to be −65 mV at the beginning of the simulation. The threshold of axonal activation was defined as the least stimulus intensity that could initiate an action potential in the axon.

When choosing the coil parameters for computation, we considered the biological relevance. Previously, a commercially available inductor was used to activate neurons *in vitro* (Bonmassar et al., 2012) and *in vivo* (Park et al., 2013). The parameters of the inductor provided by the manufacturer were used in this model, including the length of the coil (l = 0.5 mm), inductance of the coil (L = 100 nH), and resistance of the coil (R = 2 Ohm). The coil was modeled as circular in shape with *R*_*c*_ = 0.25 mm. μ_0_ = 4π × 10^−7^ H/m was the vacuum permeability.

## Results

### Activating Function Generated by a Miniature Coil and Its Dependency on Experimental Parameters

**Figure 4:**
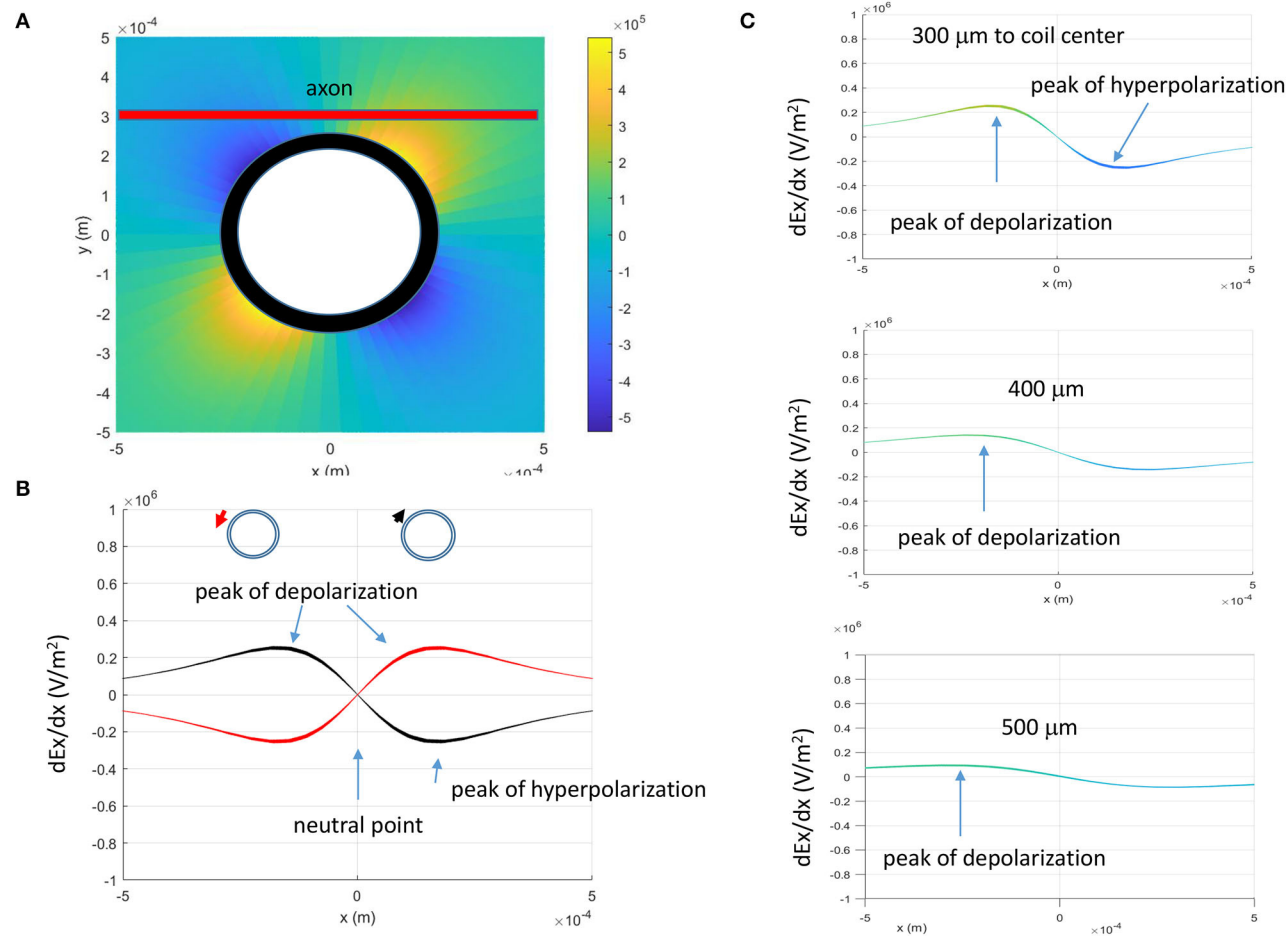
Activating function (AF, gradient of the electric field) of the miniature coil along the axon. **(A)** Activating function in the x-y plane. The thick red line represented the straight axon, 300 μm away from the center of the coil. **(B)** Activating function in the x-direction and its dependency on the direction of the induced electric fields (indicated by the red and black arrows). **(C)** Dependency of the activating function on the coil-axon distance. Peaks of depolarization moved farther away from the neutral point (AF = 0) when the coil-axon distance increased.

The activating function, defined as the gradients of the electric field along the axon (Rattay, 1986), predicts the location and speed of depolarization or hyperpolarization by the extracellular stimulation (Lee and Fried, 2017). Neural activity is generated at the location where the amount of depolarization passes a certain threshold (Lee et al., 2016, 2019). Figure 4A demonstrates the activating function distribution along an unmyelinated axon, when the distance between the coil center and the axon is 300 μm. Based on Eqs. (17) and (18), the activating function is zero when x = 0 (defined as the “neutral point”). The locations of the “peak depolarization” (virtual anode) and “peak hyperpolarization” (virtual cathode) are on each side of the “neutral point,” 173.2 μm from the “neutral point” (Eqs. 19 and 20).

The activating function is dependent on the geometric (N and *l*) and electric parameters (R and L) of the miniature coil. The time constant is defined by L/R (Eqs. 17 and 18). The coil with a longer time constant (i.e., smaller R or larger L) could provide longer membrane excitation. Because these parameters are constant for a specific coil, they do not cause shifting of the location for “peak depolarization” and “peak hyperpolarization.” Experimentally, several parameters could change. First, the direction of the coil current could switch due to human error. Second, the coil-axon distance could change due to the displacement of the coil. Third, the intensity of the stimulus could be increased to maximize the axonal response. Activating function analysis suggests that changes in these parameters could lead to the shifting of the activation location.

Prediction 1: Figure 4B demonstrates that the locations of “peak depolarization” and “peak hyperpolarization” are dependent on the direction of the magnetically induced electric field. For a single stimulation pulse, the electric field is induced during the rising phase and the falling phase of the coil current, and the axon is under a biphasic electric field stimulation (Figure 2). Therefore, any chosen point on the axon (except the “neutral point”) will experience a depolarization (or hyperpolarization) followed by a hyperpolarization (or depolarization). When the polarity of the coil current switches its sign, the location of axonal activation could shift accordingly (Figure 4B).

Prediction 2: The location of axon activation could shift if the coil-axon distance is increased (Figure 4C). Based on Eqs. (19) and (20), both the “peak depolarization” point and “peak hyperpolarization” point will move away from the “neutral point” when the coil-axon distance increases.

Prediction 3: Location of axon activation does not change with the stimulation intensity. This is because the locations of “peak depolarization” and “peak hyperpolarization” are not dependent on the intensity of the induced electric field (Eqs. 19 and 20).

### Axonal Activation by Miniature Coil With Threshold Intensity (Type I Stimulation)

A multi-compartment NEURON model of an unmyelinated axon was built to test the accuracy of these predictions (Figure 3). In the simulation, the stimulation threshold was defined as the activating function that allows the initiation of the action potential (Lee et al., 2016). Previously, an activating function of 50,000 V^2^/m (Lee et al., 2016) was capable of causing neural activation in myelinated axons. Because we used shorter pulses for the activation of a significantly larger unmyelinated axon, we expected a higher threshold for axonal activation. Indeed, when the distance between the axon and the coil center was 300 μm, the threshold was 250,000 V^2^/m for axonal activation by the short pulse (1 ms).

**Figure 5:**
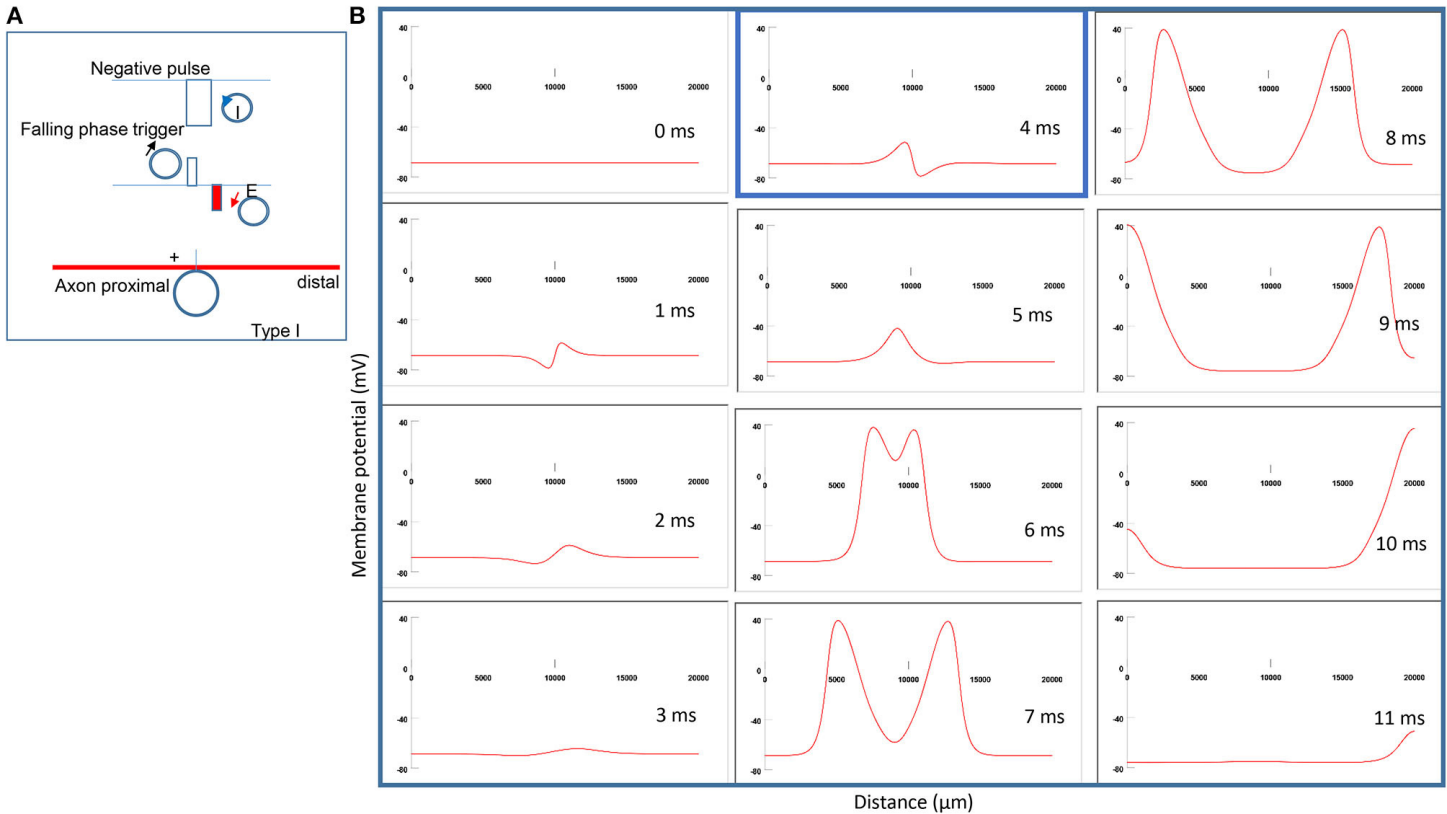
Activation of an unmyelinated axon by a miniature coil under Type I stimulation (moderate intensity). **(A)** Coil current (counterclockwise), direction of the induced electric field, and location of activation (+). The coil center was located 300 μm away from the axon. The stimulation intensity was moderate (just above the threshold). **(B)** A sequence of movie clips demonstrated the initiation and propagation of an action potential. The action potential was initiated by the falling phase of the coil current, at the location left to the neutral point, and propagated in both directions. It took less time for the action potential to propagate to the left end of the axon than to the right side. The blue box, which shows the location of activation, will be used for comparison in the following Figures 6–8.

Figure 5A demonstrates the counterclockwise coil current, the induced electric field, and the location of axonal activation. Figure 5B simulates the initiation and traveling of an action potential with a sequence of frames. When the stimulation intensity is at the threshold, one action potential is triggered at the left side of the neutral point. It takes less time for this action potential to travel to the left (proximal) side of the axon than to the right side (Figure 5B). Because the induced electric field is biphasic, the onset of coil current generates a “peak hyperpolarization” and a “peak depolarization” on the membrane (t = 2 ms, Figure 5B). However, the “peak depolarization” fails to generate an action potential. Instead, the action potential is triggered by the subsequent falling phase of the coil current, by depolarizing the membrane patch where the “peak hyperpolarization” happens during the rising phase (t = 5 ms, Figure 5B). We refer to this stimulation as the Type I stimulation. In the Type I stimulation, the axonal excitation happens at the membrane patch that experiences hyperpolarization followed by depolarization.

### Shifting of Activation Location Due to Reversal of Coil Current in Type I Stimulation

**Figure 6:**
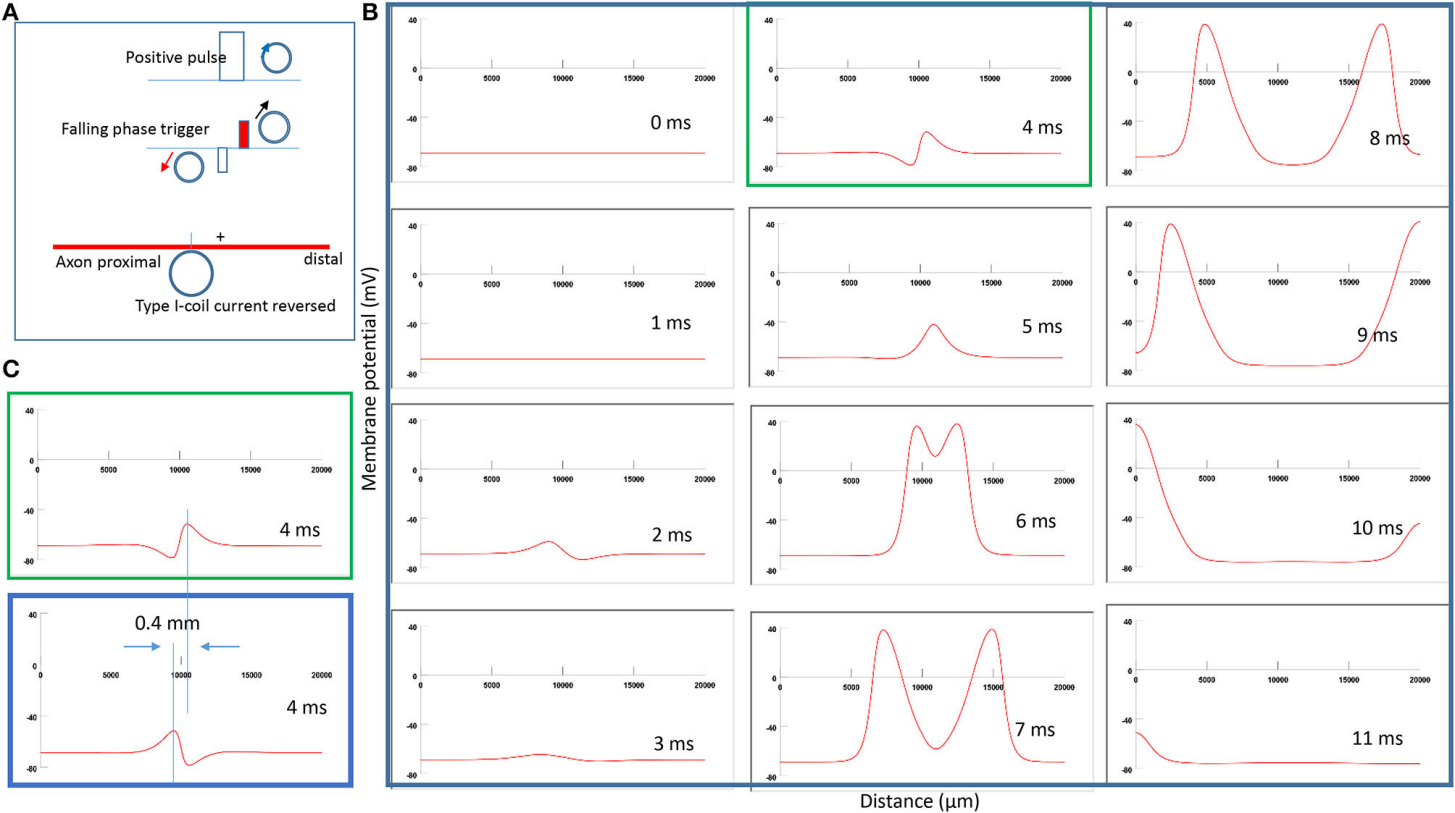
Impact of the direction of coil current on the location of activation in Type I stimulation. **(A)** A clockwise pulse current was driven by a positive pulse in the coil. “+” represents the location of activation on the axon. The action potential was triggered in the falling phase of the stimulation pulse. **(B)** A sequence of movie clips demonstrated the initiation and propagation of an action potential. The green box showed the location of activation. **(C)** Comparison of locations of activation from Figure 5 (blue box) and the green box when the coil current direction was reversed.

To test *Prediction 1*, we reversed the coil current in Figure 5A and monitored the location of axonal activation. Figure 6A demonstrates a clockwise current driven by a positive pulse in the coil. This reversal in the coil current causes a shift in the activation site from the left of the neutral point (Figure 5A) to the right side (Figure 6A). Figure 6B simulates the initiation and traveling of the action potentials with a sequence of frames. Here, the action potential requires less time to travel to the distal than to its proximal end. Figure 6C compares the locations of activation before (blue box from Figure 5B) and after (green box from Figure 6B) the coil current was reversed. In agreement with the activating function analysis (Figure 4B), when the coil current reverses its polarity, the activating point switches sides around the neutral point. The overall shift of the activation location was about 0.4 mm.

### Shift of Activation Location Due to Changes in Coil-Axon Distance

**Figure 7:**
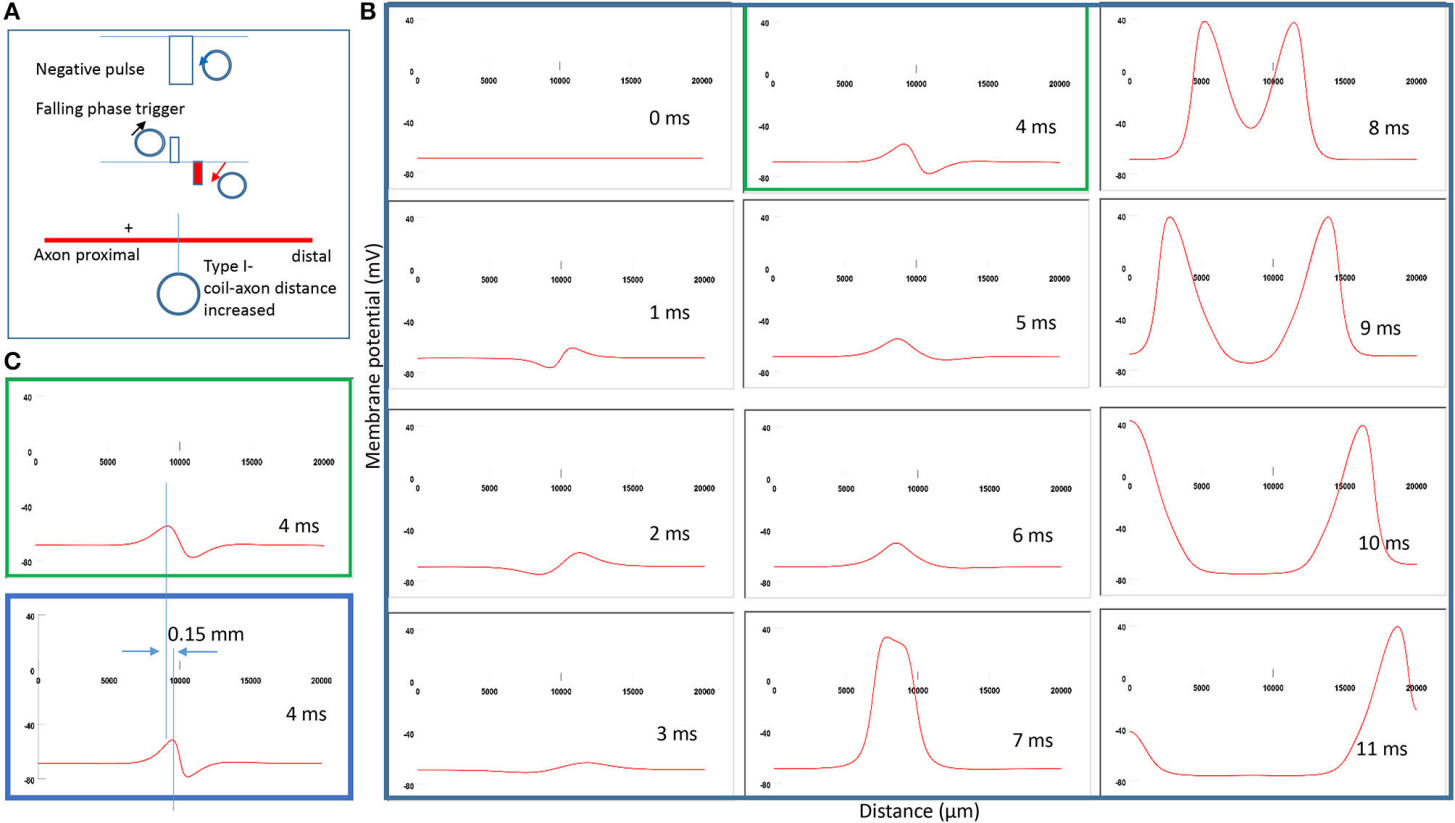
Impact of axon-coil distance on the location of excitation in Type I stimulation (falling phase activation). The coil-axon distance was increased from 300 μm (in Figure 5) to 800 μm. **(A)** Coil current, induced electric field, and location of activation (+). Stimulation intensity was adjusted to trigger action potentials. **(B)** A sequence of movie clips demonstrated the initiation and propagation of an action potential. The green box showed the location of activation. **(C)** Comparison of locations of activation from Figure 5 (blue box) and the green box when the axon-coil distance was increased.

To test *Prediction 2*, we compared the location of activation when the coil-axon distance increased from 300 to 800 μm. This corresponds to a 50–550 μm distance range between the coil edge and the tissue. Experimentally, researchers maintain the distance between the coil edge and the tissue to below 500 μm to ensure a sufficient field intensity for axonal activation [Bonmassar et al., 2012; Saha et al., 2022)]. Figure 7A demonstrates the coil current, induced electric field, and location of axonal activation (+). When the coil-axon distance increases from 300 to 800 μm, the location of activation moves proximally by about 0.15 mm. Consequently, the traveling time of the action potential to the proximal end is shortened, while the traveling time to the distal end is prolonged (Figure 7B). Figure 7C compares the locations of activation in Figure 7B (green box) and Figure 5B (blue box). In agreement with the activating function analysis (Figure 4C), when the coil moves away from the axon, the activating site moves away from the neutral point.

### Location of Activation Shifts Caused by Intensive (Type II) Stimulation

To test *Prediction 3*, the stimulating intensity gradually increases until dramatically above the threshold for Type I stimulation (named Type II stimulation).

**Figure 8:**
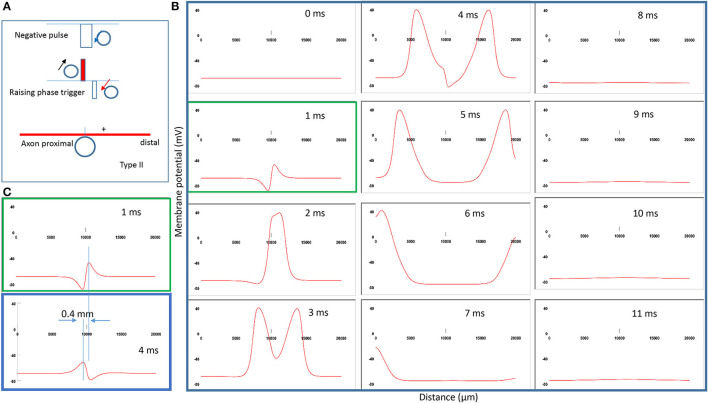
Impact of stimulation intensity on the location of activation. **(A)** Coil current, induced electric field, and location of activation (+) under a strong stimulation (Type II) with 200% threshold intensity. The action potential was triggered by the rising phase of the input pulse. **(B)** A sequence of movie clips demonstrated the initiation and propagation of the action potential by Type II stimulation. The green box showed the location of activation. **(C)** Comparison of the locations of activation from Figure 5 (blue box) and the green box when the stimulation intensity was increased.

Figure 8A demonstrates the strong counterclockwise coil current, the induced electric field, and the location of activation. Interestingly, when the stimulation intensity is extremely large (above 500,000 V^2^/m), the location of activation shifts from the left (Figure 5A) to the right (Figure 8A) of the “neutral point.” Figure 8B shows movie frames of the Type II stimulations. Here, the action potential was triggered directly by the significant membrane depolarization during the rising phase of the coil current. Figure 8C compares the different locations for activation between Type II (green box) and Type I (blue box) stimulations. The shifting of the activation site is about 0.4 mm distal to the axon, which significantly shortens the propagation of the action potential to the distal end.

Contrary to the activating function analysis, increasing the stimulation intensity did shift the location of activation, which was likely due to the complicated membrane and ion channel properties under different stimulation intensities.

### Different Ion Channel Mechanisms Underlying Type I and Type II Stimulation

**Figure 9:**
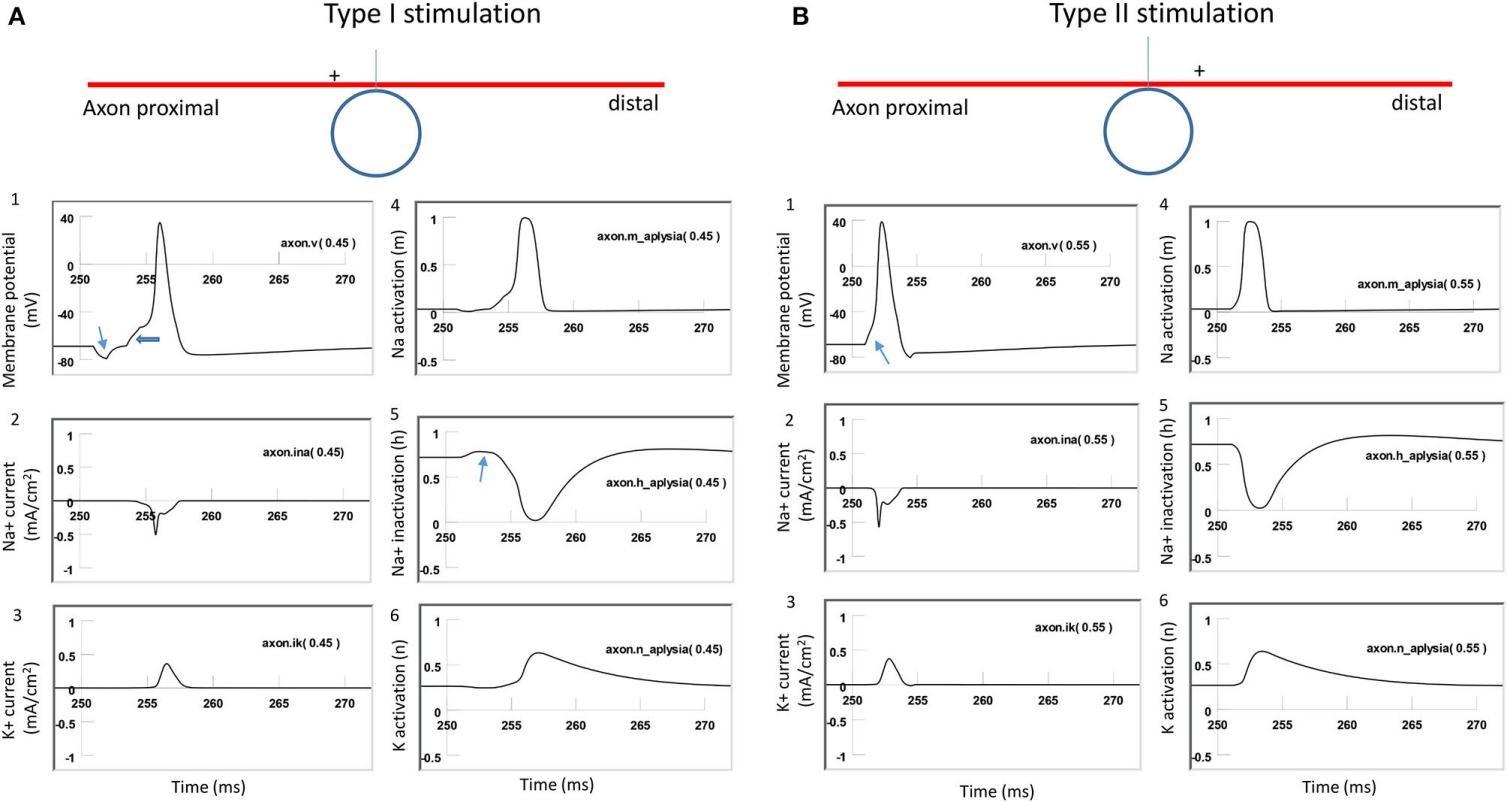
Comparison of ion channel dynamics for action potential initiation in an unmyelinated axon during Type I and Type II stimulations. Membrane potential (1), Na^+^ current (2), K^+^ current (3), sodium channel activation m (4), sodium channel inactivation h (5), and potassium channel activation n (6) were plotted at the locations where action potentials were initiated. **(A)** For Type I stimulation, the action potential was triggered at the membrane patch axon [.45]. The induced electric field during the rising phase hyperpolarized the cell membrane (thin arrow in **A1**) and removed the inactivation of the sodium channels (arrow in **A5**). Action potential was then initiated in the falling phase, which depolarized the membrane (thick arrow in **A1**). **(B)** For Type II stimulation, the action potential was triggered at the membrane patch axon [.55]. Induced electric field during the rising phase initiated the action potential simply by depolarizing the axonal membrane (arrow in **B1**) and activating the sodium channels.

To further investigate the ionic mechanism underlying action potential initiation in Type I and Type II stimulations, we studied the membrane dynamics at the locations where the action potentials were initiated. We compared the inward sodium current (INa+), outward potassium current (IK+), sodium channel activation (m) and inactivation (h) variables, and potassium channel activation (n) variables. For Type I stimulation, the action potential was initiated at the location “Axon” (0.45). Figure 9A demonstrates that this membrane patch experienced a brief hyperpolarization, followed by a depolarization, which eventually triggered an action potential. The initial hyperpolarization did not cause significant changes in the state variable m (0) and n (0.3) values. The hyperpolarization removed sodium channel inactivation and caused an increase in the h value (from 0.7 to 0.8). This led to an increase in the sodium conductance, defined as m^3^h (Hodgkin and Huxley, 1952). The subsequent depolarization caused by the falling phase was therefore sufficient in causing a large inward sodium current and generating an action potential.

For the Type II stimulation, the action potential was initiated at the location “Axon” (0.55). Figure 9B demonstrates that the membrane patch experienced a brief depolarization during the rising phase, which was significant enough to cause an action potential. The depolarization mainly activated the sodium channels by increasing the m value (from 0 to 0.3). This increased sodium channel conductance initiated the action potential. In comparison with Figure 9A (Type I stimulation), this depolarization did not alter the h value or remove the sodium channel inactivation. Therefore, Type I and Type II stimulations have different ionic mechanisms for triggering action potentials.

In summary, under weak stimulation (Type I), the local membrane was hyperpolarized during the rising phase of the coil current, which removed the inactivation of the sodium channels. This same membrane patch was then activated by the falling phase of the coil current, and an action potential was initiated. In contrast, during strong stimulation (Type II), the action potential was generated at another location, where the membrane was directly depolarized by the rising phase of the coil current. Switching the coil current or increasing the coil-axon distance could lead to the shifting of the location of activation in both Type I and Type II stimulations.

## Discussion

This study investigates the experimental factors that may cause the shifting of the excitation site on an axon under μMS. The purpose of the study is to simulate the known phenomena and generate valuable predictions. There are several major contributions of the study.

First, this study introduces the activating function for a straight, unmyelinated axon stimulated by a miniature, circular coil. In agreement with previous studies (Roth and Basser, 1990; Basser and Roth, 1991; Basser, 1994), this activating function reveals distinct anodal and cathodal regions, which have been experimentally confirmed (Nilsson et al., 1992; Maccabee et al., 1993). The derived activating function contains all major electric and geometric parameters of the miniature coil used in recent neural stimulation practice (Bonmassar et al., 2012; Skach et al., 2020; Ye and Barrett, 2021). This includes the number of loops (N), the length of the coil (l), the inductance of the coil (L), and the voltage drop across the conductance (V). Because these parameters are predetermined or measurable, the analytical expression of the activating function will be of great value in the selection of a specific miniature coil for neural stimulation.

Second, this study highlights the importance of the waveform of the induced electric field on the outcome of magnetic stimulation. In Type II stimulation, if the stimulation pulse is sufficiently wide, then both the rising and the falling phases of the induced electric field can depolarize the membrane (at different locations) and initiate action potentials. This modeling result is in agreement with a previous study (Babbs, 2014), which reported that axon segments in a sub-millimeter scale were the sites of magnetic stimulation. The axonal membrane at one end was depolarized locally during the rising phase of current in the coil. The axonal membrane at the opposite end was depolarized locally during the falling phase of current in the coil (Babbs, 2014). Similarly, Maccabee et al. (1993) also reported that a polyphasic pulse excited the nerve at two different sites on the axon by a negative first phase at one location and by a reversed second phase at the other location.

Third, this work reveals several crucial, experimentally controllable parameters on the location of excitation. In agreement with a previous study (Roth and Basser, 1990), reversing the direction of the coil current can cause shifting of the axonal activation. Therefore, a difference in the traveling time of an action potential to reach the distal end of the axon is expected when clockwise or counterclockwise monophasic current pulses are respectively used (Figure 7). Experimental data support this observation. When the median nerve in the elbow is magnetically stimulated, the site of stimulation depends on the coil current, and it shifted when the coil current is reversed (Nilsson et al., 1992). When the phrenic nerve is stimulated using a circular coil, the latency from stimulation to muscle contraction is shorter when the stimulating current flows from the proximal to the distal end of the nerve (Similowski et al., 1997).

Fourth, the results from the NEURON model and the activating function analysis are, in general, quantitatively in agreement with each other in predicting the locations of activation. However, activating function analysis is, sometimes, ineffective in accounting for the complicated interaction between the stimulus waveform and the dynamics of the ion channels, and fails to predict the shifting of the activation site caused by the increase of stimulation intensity. Specifically, the activating function analysis predicts that increasing stimulation intensity does not affect the location of depolarization (Eq. 19). However, the location of axon activation could shift dramatically when the simulation intensity varies (Figure 8). Under moderate intensity (Type I) stimulation, axon activation occurred in the area where the sodium channels were released from inactivation by membrane hyperpolarization (Figure 9A). In contrast, under high intensity stimulation (Type II), axon activation occurred in the area where sodium channels were directly activated by membrane depolarization (Figure 9B).

Finally, in this study, our interest lies in understanding the location of axonal activation by the miniature coil. We therefore constrain our detailed analysis to a single axon. However, this model is capable of analyzing numerous axons, such as those in a nerve bundle. For example, it can study the selectivity and recruitment of axons during magnetic stimulation by analyzing the orientation (distance and angle) of the individual axons to the coil plane. It can also investigate the sensitivity of the axons to magnetic stimulation, based on their morphological variations (i.e., diameter of the axon).

### Limitations of the Model and Future Directions

Due to computational complexity, several assumptions were made to simplify the modeling process. First, the extracellular electric field was always treated equally around each model compartment. Second, the extracellular electric field was not affected by the presence of the tissue. Third, the extracellular voltage generated by the membrane current was neglected. Under these assumptions, the extracellular electric field was computed without considering the existence of the tissue or its countereffect to the externally applied electric fields. Although such an approach is typical in the field (Joucla et al., 2014; Ye and Steiger, 2015), it must be noted that these simplifications can potentially cause underestimation of the field generated by the miniature coil and introduce errors (McIntyre et al., 2004; Lee and Grill, 2005).

Several limitations are inherent in the NEURON model. According to previous studies (Basser et al., 1992; Lu et al., 2008), the axon was modeled as a straight cylinder without considering nerve undulation and the bending of the axon for local activation (Abdeen and Stuchly, 1994). More complicated neural morphology could be modeled with a numerical method, such as finite element modeling (Joucla et al., 2014). The model assumed that only the electric field varying in the longitudinal direction could activate the axon, but not in the field in the orthogonal directions. Recent theoretical studies suggested that besides the longitudinal field, which defines the activating function, the orthogonal field components to the axon could also contribute to axon activation (Ye et al., 2011; Wang et al., 2018a,b). Finally, the unmyelinated axon model did not consider other neurons and axons in the proximate distance of the modeled axon, which could perturb the local field (Pucihar et al., 2006). The Hodgkin-Huxley-based ion channel mechanism did not include several ion channels, such as Ca^2+^ channels and A-type K^+^ channels that are essential for neural excitability (Tan et al., 2013). The model did not include stimulation-induced ion gradient changes, such as excessive K^+^ ionic accumulation in the extracellular space, which has been observed during electric stimulation (Bikson et al., 2001; Lian et al., 2003). Further modeling work, driven by the new experimental data, shall address these limitations in the current model.

This model was based on the activation of an unmyelinated axon by the micro-coil, rather than using the myelinated axon model. This arrangement was to serve the goal of comparing the locations of axon activation using both the activating function analysis and the multi-compartment NEURON model. In unmyelinated axons, the location of depolarization is theoretically the site of action potential initiation. In myelinated axons, the action potentials are normally observed in the nodes of Ranvier. The internode interval in myelinated axons could be as large as 700 μm, as seen in the mice sciatic nerve (Villalon et al., 2018), a dimension nearly comparable to the size of the micro-coil. Therefore, the presence of the myelin sheath could significantly offset the location of the activation site. Using myelinated axon modeling could cause inconsistencies between the (activating function predicated) location of depolarization and the (NEURON modeled) initiation of the action potential. Nevertheless, it is worth to model the stimulation of myelinated axons with the miniature coil, as it is essential for the development of μMS technology to stimulate axons in the central nervous system. Numerous methods established in this work could be applied to study the myelinated axon stimulation with μMS.

This study models the effect of μMS driven by pulsatile stimuli as previously reported experimentally (Bonmassar et al., 2012; Lee et al., 2016). Several studies in the field also used sine waves as the stimulus for axon stimulation (Lee et al., 2019; Saha et al., 2022). The induced electric field is the time derivative of the coil waveform. Therefore, the induced electric field is a triphasic waveform under sine wave stimulation (Lee et al., 2016, 2019). It is foreseeable that this waveform will cause oscillation to the local axonal membrane and possible preconditioning of the ion channels for membrane excitation, as introduced in this study (Figure 9A). The ultimate outcome of the stimulation is dependent on the interaction between this triphasic waveform and the channel dynamics, which can be further investigated with the multi-compartment model introduced in this study.

This modeling study did not consider the potential thermal effects of the coil stimulation, as it did not occur when a single, short pulse was used for neural stimulation (Bonmassar et al., 2012; Lee et al., 2016). When trains of high-frequency pulses are used for neural stimulation, however, the miniature coil could potentially cause thermal effects to the neural tissue (Skach et al., 2020). Significant heating effects could cause damage to the coil or introduce irreversible tissue damage. Modeling results from this study are, therefore, limited to non-thermal stimulation. Future modeling studies must consider the effect of temperature on the initiation and propagation of the action potentials.

### Implications to the Consistency and Biocompatibility of Miniature Coil Technology

Regardless of the limitations of the model, this study suggests that the miniature coil could provide improved consistency over traditional stimulation protocols using an implanted metal electrode.

While electrodes have been widely implanted to stimulate neural tissues, maintaining consistency in stimulation is challenging. This is because the electric field produced by an electrode is influenced by the inhomogeneity of the medium surrounding the nerve and electrodes (Rattay, 1989, 2008). Tissue homogeneity and anisotropy could affect the excitation threshold (Hyodo et al., 2009) and distribution of the induced electric field (Ye and Steiger, 2015). For a point electrode, the activating function depends on ρ_*e*_ or the conductivity of the medium surrounding the electrode (Rattay, 1986). Any changes in ρ_*e*_ could cause inconsistency in the activating function for a chronically implanted electrode. This includes inflammation reaction of the tissue (Kim et al., 2004), glial scar formation around the electrode (Polikov et al., 2005; Grill et al., 2009), oxidization of the electrode, and other bioelectric changes around the electrode, such as accumulation of extracellular potassium during deep brain stimulation (Shin and Carlen, 2008).

In comparison, under miniature coil stimulation, the activating function of the coil is not dependent on the tissue properties around the coil (Eqs. 17 and 18). The consistency of coil stimulation will not be affected by physiological and pathological changes in the microscopic environment surrounding the coil (Golestanirad et al., 2018). This property also allows the miniature coil to be coated with a biocompatible material for long-term implantation. Unlike the electric fields generated by the electrodes, magnetic fields pass readily through the coating of biocompatible materials, even under severe encapsulation; therefore, their efficacy is not diminished.

### Implications of Improving Specificity of Neural Activation by the Miniature Coil

Achieving focal stimulation to the nerve tissue is the ultimate goal of the novel μMS technology. Previously, when the coil size was significantly larger than the length of the axon, the gradients of the induced electric field were purely dependent on the coil. It is therefore difficult to identify a single point for activation. The location of activation is largely defined by the sharp curvature of the nerve, which causes a dramatic increase of the field gradient in a local area. For the sub-millimeter-sized coil, the local electric field is curved, which ensures local depolarization of certain axon segments and allows for better selectivity in activating short axons. The results from this study provide several insights to further improve the specificity of neural stimulation by μMS technology.

First, because the activating function is defined as the electric field gradient along the axon, achieving a quantitatively great activating function is key to activating axons (Ye and Steiger, 2015). This can be done by implanting the coil close to the targeted nerve to form a local field gradient. The coil can also be customized with sharp angles to generate a large local field gradient, which maximizes the local activating function (Lee et al., 2016, 2019).

Second, orientation between the coil and the axon plays a significant role in axonal activation, as demonstrated with Eqs. (17), (18), and (21). Indeed, when the induced electric field is parallel to the soma-axon axial, a single pulse generates trains of action potentials in retinal ganglion cells. When the induced electric field is perpendicular to the soma-axon axial, coil stimulation has limited effectiveness in eliciting action potentials in these neurons (Bonmassar et al., 2012). In another example, Lee and Fried (2017) showed that layer V pyramidal neurons (PNs) are strongly activated by the μMS, as long as a great field gradient along the neural process is guaranteed.

Third, care must be taken to avoid the unwanted shifting of the location of neural excitation during μMS. It is essential to control the direction of the coil current (Figure 6), avoid possible displacement of the coil (Figure 7), and maintain a consistent stimulation intensity (Figure 8).

Fourth, care should be taken in designing the waveforms that drive the miniature coil for neural activation, as the shape and pulse width of the coil current may play significant roles in the location of the activation. In electrode stimulation, charge balanced waveforms are preferable, as they can avoid possible corrosion of the electrodes caused by irreversible redox reactions (Merrill et al., 2005). In comparison, μMS stimulation has the advantage in applying many waveforms to drive the miniature coil. In this model, the waveform of the induced electric field led to complicated dynamics of local membrane depolarization and hyperpolarization, which determine the ultimate location of activation. In Type I stimulation with moderate stimulation intensity, pulse width was found to play important roles in releasing the inactivation in the sodium channels for later activation (Figure 9A). Too long or too short of a pulse width could compromise the ion channel mechanisms for axonal activation. Several findings support this notion. In a simulation study, the coil current waveform was found to be the most important parameter. Biphasic coil current has a lower threshold than monophasic coil current in axonal activation (Hyodo et al., 2009). In an *in vivo* experiment, where micromagnetic fields were used to activate the inferior colliculus neurons, the authors found that certain pulse widths generated a stronger response than others (Park et al., 2013). This study provides a platform to evaluate the impact of various waveforms on the outcome of μMS.

Finally, because the activating function can only predict the location of depolarization (but not activation), merely computing the electric field and its gradient in a nerve tissue is not sufficient to predict the location of activation. In this model, intensive stimulation causes direct activation of the axon in a specific location (Figure 8), while moderate stimulation activates the axon at a different location (Figure 4). This study highlights the importance of understanding the complex biophysics properties of the cell membrane and its interaction with the field during μMS (Ye and Steiger, 2015). In this regard, the computer simulation with multi-compartment modeling is extremely powerful to include these biophysical properties and produce more accurate model predictions.

## Conclusions

We demonstrate the potential of the miniature coil in axonal activation and investigate the ion channel mechanisms underlying this activation. Notably, in some cases, the location of axonal activation shifts due to experimental errors. The results from this study can be used to guide future animal experiments, as well as to optimize the design of miniature coils in clinical applications, to further improve the outcome of stimulation by this novel neuromodulation method. The μMS technology could be an interesting alternative to the conventional implanted electrodes for neuromodulation.

## Data Availability Statement

The original contributions presented in the study are included in the article/Supplementary Material, further inquiries can be directed to the corresponding author.

## Author Contributions

HY conducted the biophysics modeling and the NEURON simulation, and wrote the manuscript.

## Funding

This research was funded by the Research Support Grant from Loyola University Chicago.

## Conflict of Interest

The author declares that the research was conducted in the absence of any commercial or financial relationships that could be construed as a potential conflict of interest.

## Publisher's Note

All claims expressed in this article are solely those of the authors and do not necessarily represent those of their affiliated organizations, or those of the publisher, the editors and the reviewers. Any product that may be evaluated in this article, or claim that may be made by its manufacturer, is not guaranteed or endorsed by the publisher.

## Supplementary Material

NEURON Code and descriptions:

1. Aplysia.mod: mod file that defines sodium, potassium, and leakage channels.
2. Init.hoc: main program that ran the simulation.
3. Axonmodel.hoc: defines the unmyelinated axon.
4. Biphasic.hoc: defines spatial and temporal feathers of the stimuli; the induced electric field by the miniature coil is biphasic.
5. plot_e_extra.ses: session file for the display of simulation results.
6. rig.ses: session file for the display of simulation results.
