# Supplementary material for: Finding the Location of Axonal Activation by a Miniature Magnetic Coil
Source: Front Comput Neurosci. 2022 Jun 29;16:932615. doi: 10.3389/fncom.2022.932615 (PMC9276924; doi:10.3389/fncom.2022.932615)
Supplement: Supplementary file 1 [file Data_Sheet_1.DOCX]

TITLE aplysia.mod sodium, potassium, and leak channels

COMMENT

This is the modification of the original Hodgkin-Huxley treatment for the set of sodium, potassium, and leakage channels to descript unmyelinated axon in Aplysia.

[Skach J, Conway C, Barrett L, Ye H. Axonal blockage with microscopic magnetic stimulation. Scientific reports. 2020;10(1):18030. doi: 10.1038/s41598-020-74891-3. PubMed PMID: 33093520; PubMed Central PMCID: PMC7582966.]

Membrane voltage is in absolute mV and has been reversed in polarity

from the original HH convention and shifted to reflect a resting potential of -65 mV. Remember to set celsius=6.3 (or whatever) in your HOC file.

ENDCOMMENT

UNITS {

(mA) = (milliamp)

(mV) = (millivolt)

(S) = (siemens)

}

? interface

NEURON {

SUFFIX aplysia

USEION na READ ena WRITE ina

USEION k READ ek WRITE ik

NONSPECIFIC_CURRENT il

RANGE gnabar, gkbar, gl, el, gna, gk

GLOBAL minf, hinf, ninf, mtau, htau, ntau

}

PARAMETER {

gnabar = 0.12 (S/cm2) <0,1e9>

gkbar = 0.036 (S/cm2) <0,1e9>

gl = .00028 (S/cm2) <0,1e9>

el = -65 (mV)

}

STATE {

m h n

}

ASSIGNED {

v (mV)

celsius (degC)

ena (mV)

ek (mV)

gna (S/cm2)

gk (S/cm2)

ina (mA/cm2)

ik (mA/cm2)

il (mA/cm2)

minf hinf ninf

mtau (ms) htau (ms) ntau (ms)

}

LOCAL mexp, hexp, nexp

? currents

BREAKPOINT {

SOLVE states METHOD cnexp

gna = gnabar*m*m*m*h

ina = gna*(v - ena)

gk = gkbar*n*n*n*n

ik = gk*(v - ek)

il = gl*(v - el)

}

INITIAL {

rates(v)

m = minf

h = hinf

n = ninf

}

? states

DERIVATIVE states {

rates(v)

m' = (minf-m)/mtau

h' = (hinf-h)/htau

n' = (ninf-n)/ntau

}

LOCAL q10

? rates

PROCEDURE rates(v(mV)) { :Computes rate and other constants at current v.

:Call once from HOC to initialize inf at resting v.

LOCAL alpha, beta, sum

TABLE minf, mtau, hinf, htau, ninf, ntau DEPEND celsius FROM -100 TO 100 WITH 200

UNITSOFF

q10 = 3^((celsius - 6.3)/10)

:"m" sodium activation system

alpha = .1 * vtrap(-(v+40),10)

beta = 4 * exp(-(v+65)/18)

sum = alpha + beta

mtau = 1/(q10*sum)*3.0

minf = alpha/sum

:"h" sodium inactivation system

alpha = .07 * exp(-(v+65)/20)

beta = 1 / (exp(-(v+35)/10) + 1)

sum = alpha + beta

htau = 1/(q10*sum)*1.7

hinf = alpha/sum

:"n" potassium activation system

alpha = .01*vtrap(-(v+55),10)

beta = .125*exp(-(v+65)/80)

sum = alpha + beta

ntau = 1/(q10*sum)*5.6

ninf = alpha/sum

}

FUNCTION vtrap(x,y) { :Traps for 0 in denominator of rate eqns.

if (fabs(x/y) < 1e-6) {

vtrap = y*(1 - x/y/2)

}else{

vtrap = x/(exp(x/y) - 1)

}

}

UNITSON

// File name: init.hoc

load_file("nrngui.hoc")

load_file("Axonmodel.hoc")

load_file("rig.ses")

load_file("Biphasic_stim.hoc")

load_file("plot_e_extra.ses")

celsius=20

// File name: Axonmodel.hoc. Defines an unmyelinated axon

create axon

access axon

nseg = 200

L = 20000

diam = 15

insert aplysia

// File name: Biphasic_stim.hoc

// create the basic stimulus time course for Extracellular stimulation by // a miniature coil. The induced electric field is a bi-phasic signal.

// The spatial distribution of the induced electric field alone the axon // is in equations (20 and (27), for the rising phase and falling phase // in the induced electric field, respectively.

amplitude = 1

factor=1

delay = 250 // for long delay, set the m, h,n parameters better

duration = 5

xcoil = 10000 // location of the coil, middle of a 20000 um axon

ycoil = -300 // coil’s distance to the axon

Rcoil = 0.25e-3

Ncoil=20

lengthcoil=0.5e-3

Lcoil=100e-9

U0=12.56e-7

CONcoil=(Rcoil*Rcoil/2)*(U0*Ncoil)/(Lcoil* lengthcoil)

objref tvec, pvec

// tvec will hold the stimulus sample times (X NUM of pulses)

// pvec will be a 1 ms pulse of with amplitude PMAX- HUIHUI

NUMPTS = 5 //5 ms pulse duration.

NUMPULSE=duration/5 // duration stimulation converted into # of pulses

TOTALNUMPTS= NUMPTS* NUMPULSE + delay

PMAX = amplitude // COIL voltage that can elicit spikes.

tvec = new Vector(TOTALNUMPTS)

pvec = new Vector(TOTALNUMPTS)

for j=0,delay-1 { tvec.x[j]=j pvec.x[j]=0 }

for i=0, NUMPULSE-1 {

{ tvec.x[NUMPTS*i+0+delay]=0+NUMPTS*i+j pvec.x[NUMPTS*i+0+delay]=0 }

{ tvec.x[NUMPTS*i+1+delay]=1+NUMPTS*i+j pvec.x[NUMPTS*i+1+delay]=-PMAX }

{ tvec.x[NUMPTS*i+2+delay]=2+NUMPTS*i+j pvec.x[NUMPTS*i+2+delay]=0 }

{ tvec.x[NUMPTS*i+3+delay]=3.5+NUMPTS*i+j pvec.x[NUMPTS*i+3+delay]=PMAX }

{ tvec.x[NUMPTS*i+4+delay]=4.5+NUMPTS*i+j pvec.x[NUMPTS*i+4+delay]=0 }

}

print " "

print "tvec"

tvec.printf

print " "

print "pvec"

pvec.printf

// drive e_extracellular at each internal node of the model

// with a voltage that has the time course specified by pvec, tvec

forall insert extracellular

objref veclist // will hold all the stim Vectors

proc setstim() { localobj tmpvec

veclist = new List()

forall {

for (x, 0) { // iterate over internal nodes only

// specify the time course of extracellular potential

// at this location

tmpvec = pvec.c

tmpvec.mul((amplitude* factor*CONcoil)*atan(((1-x)*L-xcoil)/ycoil))

tmpvec.play(&e_extracellular(x), tvec)

veclist.append(tmpvec)

}

}

}

setstim()

xpanel("Stimulus Parameters")

xvalue("Coil Stimulus Ampl (V)", "amplitude", 1, " setstim() run()", 1)

xpanel()

plot_e_extra.ses: session file for the display of simulation results.

{load_file("nrngui.hoc")}

objectvar save_window_, rvp_

objectvar scene_vector_[6]

objectvar ocbox_, ocbox_list_, scene_, scene_list_

{ocbox_list_ = new List() scene_list_ = new List()}

{pwman_place(0,0,0)}

//Begin I/V Clamp Electrode

{

load_file("electrod.hoc")

}

{

ocbox_=new Electrode(0)

execute("can_locate=1 sec=\"axon\" xloc=0.0225 locate(0)", ocbox_)

execute("vc.dur[0]=0.1 vc.amp[0]=-65", ocbox_)

execute("vc.dur[1]=2.5 vc.amp[1]=10", ocbox_)

execute("vc.dur[2]=100 vc.amp[2]=-65", ocbox_)

execute("stim.del=0.1 stim.dur=10 stim.amp=2", ocbox_)

execute("vcsteps=5", ocbox_)

execute("samp=stim.amp store_vclamp() glyph()", ocbox_)

ocbox_ = ocbox_.v1

ocbox_.map("I/V Clamp Electrode", 938, 21, 222.3, 364.5)

}

objref ocbox_

//End I/V Clamp Electrode

{

xpanel("RunControl", 0)

v_init = -65

xvalue("Init","v_init", 1,"stdinit()", 1, 1 )

xbutton("Init & Run","run()")

xbutton("Stop","stoprun=1")

runStopAt = 5

xvalue("Continue til","runStopAt", 1,"{continuerun(runStopAt) stoprun=1}", 1, 1 )

runStopIn = 1

xvalue("Continue for","runStopIn", 1,"{continuerun(t + runStopIn) stoprun=1}", 1, 1 )

xbutton("Single Step","steprun()")

t = 100

xvalue("t","t", 2 )

tstop = 100

xvalue("Tstop","tstop", 1,"tstop_changed()", 0, 1 )

dt = 0.025

xvalue("dt","dt", 1,"setdt()", 0, 1 )

steps_per_ms = 40

xvalue("Points plotted/ms","steps_per_ms", 1,"setdt()", 0, 1 )

screen_update_invl = 0.05

xvalue("Scrn update invl","screen_update_invl", 1,"", 0, 1 )

realtime = 92.54

xvalue("Real Time","realtime", 0,"", 0, 1 )

xpanel(1,157)

}

{

save_window_ = new Graph(0)

save_window_.size(0,100,-80,40)

scene_vector_[2] = save_window_

{save_window_.view(0, -80, 100, 120, 404, 21, 300.6, 200.8)}

graphList[0].append(save_window_)

save_window_.save_name("graphList[0].")

save_window_.addexpr("v(.5)", 1, 1, 0.8, 0.9, 2)

}

{

save_window_ = new Graph(0)

save_window_.size(0,100,-80,40)

scene_vector_[3] = save_window_

{save_window_.view(0, -80, 100, 120, 418, 370, 300.6, 200.8)}

flush_list.append(save_window_)

save_window_.save_name("flush_list.")

objectvar rvp_

rvp_ = new RangeVarPlot("v")

axon rvp_.begin(0)

axon rvp_.end(1)

rvp_.origin(0)

save_window_.addobject(rvp_, 2, 1, 0.8, 0.9)

}

{

xpanel("Movie Run", 0)

xbutton("Init & Run","movierun()")

movie_frame_dur_ = 0.01

xvalue("Seconds per step","movie_frame_dur_", 1,"", 0, 1 )

xpanel(12,596)

}

{

save_window_ = new Graph(0)

save_window_.size(0,20000,0,10000)

scene_vector_[4] = save_window_

{save_window_.view(0, 0, 20000, 10000, 883, 478, 300.6, 200.8)}

flush_list.append(save_window_)

save_window_.save_name("flush_list.")

objectvar rvp_

rvp_ = new RangeVarPlot("e_extracellular")

axon rvp_.begin(0)

axon rvp_.end(1)

rvp_.origin(0)

save_window_.addobject(rvp_, 2, 1, 0.451757, 0.909585)

}

objectvar scene_vector_[1]

{doNotify()}

rig.ses: session file for the display of simulation results.

{load_file("nrngui.hoc")}

objectvar save_window_, rvp_

objectvar scene_vector_[5]

objectvar ocbox_, ocbox_list_, scene_, scene_list_

{ocbox_list_ = new List() scene_list_ = new List()}

{pwman_place(0,0,0)}

{

xpanel("RunControl", 0)

v_init = -65

xvalue("Init","v_init", 1,"stdinit()", 1, 1 )

xbutton("Init & Run","run()")

xbutton("Stop","stoprun=1")

runStopAt = 5

xvalue("Continue til","runStopAt", 1,"{continuerun(runStopAt) stoprun=1}", 1, 1 )

runStopIn = 1

xvalue("Continue for","runStopIn", 1,"{continuerun(t + runStopIn) stoprun=1}", 1, 1 )

xbutton("Single Step","steprun()")

t = 0

xvalue("t","t", 2 )

tstop = 5

xvalue("Tstop","tstop", 1,"tstop_changed()", 0, 1 )

dt = 0.025

xvalue("dt","dt", 1,"setdt()", 0, 1 )

steps_per_ms = 40

xvalue("Points plotted/ms","steps_per_ms", 1,"setdt()", 0, 1 )

screen_update_invl = 0.05

xvalue("Scrn update invl","screen_update_invl", 1,"", 0, 1 )

realtime = 0

xvalue("Real Time","realtime", 0,"", 0, 1 )

xpanel(4,109)

}

{

save_window_ = new Graph(0)

save_window_.size(0,5,-80,40)

scene_vector_[2] = save_window_

{save_window_.view(0, -80, 5, 120, 296, 109, 300.48, 200.32)}

graphList[0].append(save_window_)

save_window_.save_name("graphList[0].")

save_window_.addexpr("v(.5)", 1, 1, 0.8, 0.9, 2)

}

{

save_window_ = new Graph(0)

save_window_.size(0,100,-80,40)

scene_vector_[3] = save_window_

{save_window_.view(0, -80, 100, 120, 296, 375, 300.48, 200.32)}

flush_list.append(save_window_)

save_window_.save_name("flush_list.")

objectvar rvp_

rvp_ = new RangeVarPlot("v")

axon rvp_.begin(0)

axon rvp_.end(1)

rvp_.origin(0)

save_window_.addobject(rvp_, 2, 1, 0.8, 0.9)

}

{

xpanel("Movie Run", 0)

xbutton("Init & Run","movierun()")

movie_frame_dur_ = 0.01

xvalue("Seconds per step","movie_frame_dur_", 1,"", 0, 1 )

xpanel(4,511)

}

objectvar scene_vector_[1]

{doNotify()}
